# The Imperial Joint Line Congruency Measurement is a valuable tool in total knee arthroplasty

**DOI:** 10.1371/journal.pone.0257325

**Published:** 2021-09-10

**Authors:** Ravi Popat, Kieran Dhillon, Piyush Mahapatra, Hasaan Khan, Dinesh Nathwani

**Affiliations:** 1 Department of Trauma & Orthopaedic Surgery, Imperial College Healthcare NHS Trust, London, United Kingdom; 2 Imperial College London, London, United Kingdom; Paracelsus Medizinische Privatuniversitat - Nurnberg, GERMANY

## Abstract

**Background:**

Preservation of joint line height is an important factor in post-operative function after Total Knee Arthroplasty (TKA). This is the first study investigating the reliability of the novel Imperial Joint Line Congruency Measurement (IJLCM) technique for the assessment of joint line height using plain radiographs.

**Methods:**

The reliability of two techniques used to measure joint line height on pre-operative and post-operative plain radiographs is presented. 120 patients that underwent TKA from 6 different international centres were included. Measurements were performed using each technique by two senior orthopaedic surgeons at two different timepoints (test-retest). Two undergraduate medical students performed joint line measurements using the most reproducible of the two techniques on 40 pre-operative and post-operative images to establish the reliability of the measurement technique.

**Results:**

The IJLCM demonstrated an average absolute difference of 1.83mm (CI 1.56–2.10mm) and excellent inter and intra-rater reliability between senior orthopaedic surgeons (>0.92 (CI 0.88–0.94) when measuring joint line height on plain radiographs. Overall Crohnbach’s alpha over 0.92 confirmed internal consistency. Measurements performed using the control technique as previously described by Figgie et al. had an average absolute difference of 5.75mm (5.17–6.32mm). Comparison of measurements by senior orthopaedic surgeons and medical students using the IJLCM technique with ANOVA and student’s t-test demonstrated acceptable agreement and inter-rater reliability of >0.92 (0.87–0.95).

**Conclusion:**

This study shows excellent accuracy, precision, and reliability of the novel IJLCM technique. Furthermore, excellent agreement between senior orthopaedic surgeons and medical students when using the IJLCM could be shown. The IJLCM technique is reliable for joint line assessment.

## Introduction

Preservation of the anatomical joint line height has been shown to be an important factor in post-operative function following Total Knee Arthroplasty (TKA) [1]. Instability in the knee can occur with as little as 5mm alteration in joint line height [2, 3]. In the long term, stability can affect survival of the implant, functional outcomes and patient-reported outcome measures [2].

Post-operative range of movement has been reported to be affected by alterations in joint line height. A 2mm change in height has been shown to have a clinically significant impact [4, 5].

Furthermore, displacement of the joint line by 3mm has been shown to alter the biomechanics of the patellofemoral joint and function of the quadriceps muscle [6–8]. Displacement of the joint line distally can result in pain and subluxation [8]. Increasing the joint line height leads to impingement of the patella and patella tendon [1, 9].

Measurement of joint line height has previously been performed using plain radiographs. Figgie et al. [10] and Kawamura et al. [11] have provided measurement techniques that have been used in subsequent studies [12–19]. The control technique as previously described by Figgie et al. [10] has been referred to as the gold standard for radiological measurement [12]. The technique involves measuring the distance from the tibial tubercle to the tibial plateau, along a line that is perpendicular to the joint line on lateral radiographs. The tibial tubercle can vary in shape and size between patients. Reliably identifying a single point to represent the tibial tubercle can be difficult. With potential inconsistency regarding the starting point from which to perform a measurement, the reliability of the technique is called in to question.

Snider et al. [12] modified the technique described by Kawamura et al. [11]. The technique involves measuring the distance from the top of the fibula head to the tibial plateau on AP radiographs. Snider et al. [12] suggest that accurate restoration of joint line height does not impact functional outcomes. The authors failed to correct for magnification error between pre-operative and post-operative images, and also failed to account for cartilage thickness.

The aim of this study was to assess the reliability of the Imperial Joint Line Congruency Measurement (IJLCM) technique and the control technique [10]. The null hypothesis for this study was that there is no difference in reliability between using the IJLCM and the control technique. There was also no difference between accuracy and precision of measurements performed by senior orthopaedic surgeons and medical students.

## Methods

Pre- and post-operative plain radiographs (with orthogonal views consisting of anteroposterior [AP] and lateral projections) for 120 patients operated on for a TKA were analysed retrospectively. The patients’ radiographs were taken between 1 January and 1 October 2019. Patients were recruited for 6 centres in 5 countries (Imperial College Healthcare NHS Trust, London, UK; London North West University Healthcare NHS Trust, London, UK; Hannover Medical School, Annastift Hospital, Hannover, Germany; St Trudo Hospital, Sint Truiden, Belgium; Busmaed Paardevlei Private Hospital, Cape Town, South Africa, Mediclinic City Hospital, Dubai, UAE). Surgery was performed by 12 knee surgeons. Images were accessed on 1 December 2019. All images were fully anonymised before observers were granted access to them. The images were analysed with computer software OsiriX®(Pixmeo, Bernex, Switzerland).

### Exclusion criteria

Images where calibration of measurements could not be performed were excluded from the study. Other exclusion criteria were patients with a fixed flexion deformity (this would affect the measurement of joint line height), images where the most proximal point on the fibula was not visible and images where the tibial metaphysis and diaphysis were not visible (where the observers could not identify the long axis of the tibia)

### Observers

All images were reviewed by 2 senior orthopaedic surgeons. Both observers performed measurements using the control and the IJLCM techniques separately. Both surgeons measured each set of plain radiographs twice, with at least two weeks in between the sets of measurements. Two undergraduate medical students also performed joint line measurements using the most reproducible of the two techniques on 40 pre-operative and post-operative images. The medical students had no previous experience of performing measurement on plain radiographs. The students measured each set of plain radiographs twice, with two weeks between each set of measurements.

### Radiographic quality

All patients underwent weightbearing AP and lateral plain radiographs of the affected knee joint. Radiographs were taken pre-operatively and post-operatively. A true lateral radiograph involved both femoral condyles being superimposed, giving the appearance of a single femoral condyle [20]. All pre-operative radiographs were calibrated using calibration discs. Post-operative radiographs were calibrated using the keel of the tibial component, which was of a pre-determined length. All radiographic images were reviewed by both orthopaedic surgeons to ensure adequate image quality for inclusion in the study.

### Measurement techniques

**Fig 1:**
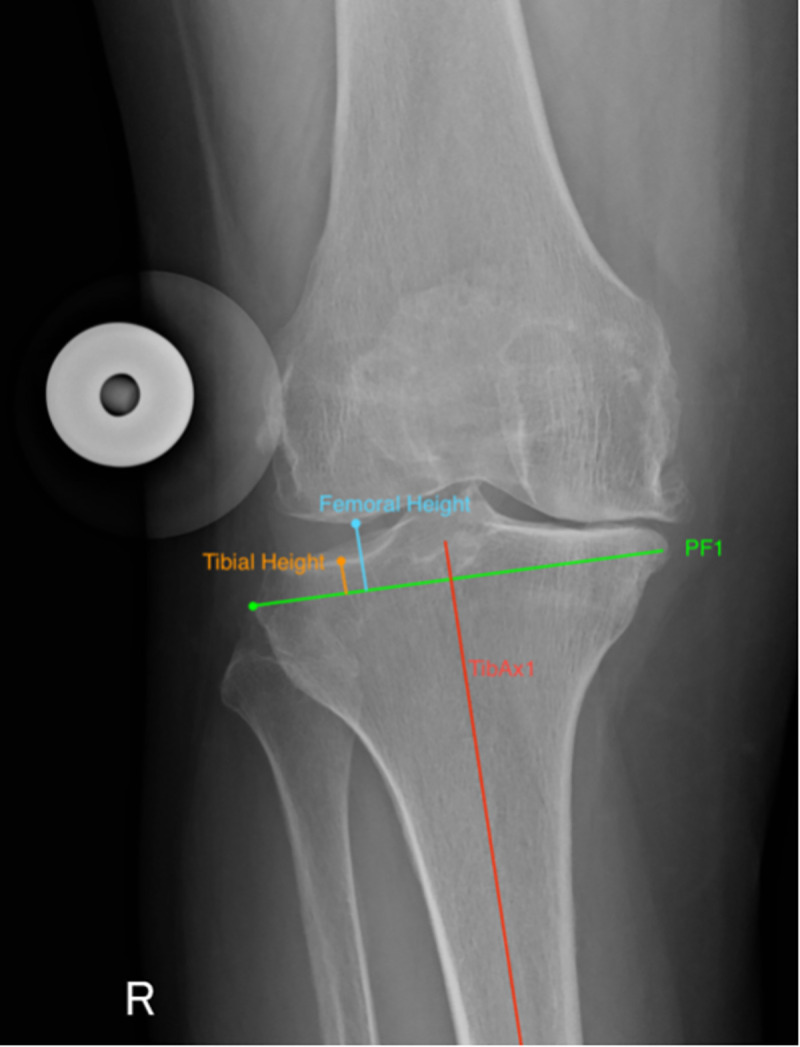
Pre-operative IJLCM technique. Assessment of pre-operative joint line height calculated as the average value of the tibial height and femoral height on the least affected side.

Pre- and post-operative joint line height measurements were performed using two different techniques:

1. Control technique described by Figgie et al. (1986): Joint line height was assessed on lateral weight bearing radiographs. The distance from the tibial tubercle to the tibial plateau, along a line that is perpendicular to the joint line was measured.
2. IJLCM technique (Fig 1): Joint line height was assessed on calibrated pre-operative weightbearing AP radiographs. The intra-medullary axis of the tibia (TibAx1) was initially established. A line perpendicular to TibAx1 at the level of the most proximal point of the proximal fibula (PF1) was drawn. *Tibial height*: the length of a line drawn parallel to the TibAx1, connecting the most proximal point on the tibial plateau (on the least affected side) to PF1 was measured. *Femoral condyle height*: the length of a line drawn parallel to the TibAx1, connecting the most distal point on the femoral condyle (on the least affected side) to PF1 was measured. Cartilage thickness was determined by calculating the average between the tibial height and femoral condyle height on the least affected side.

**Fig 2:**
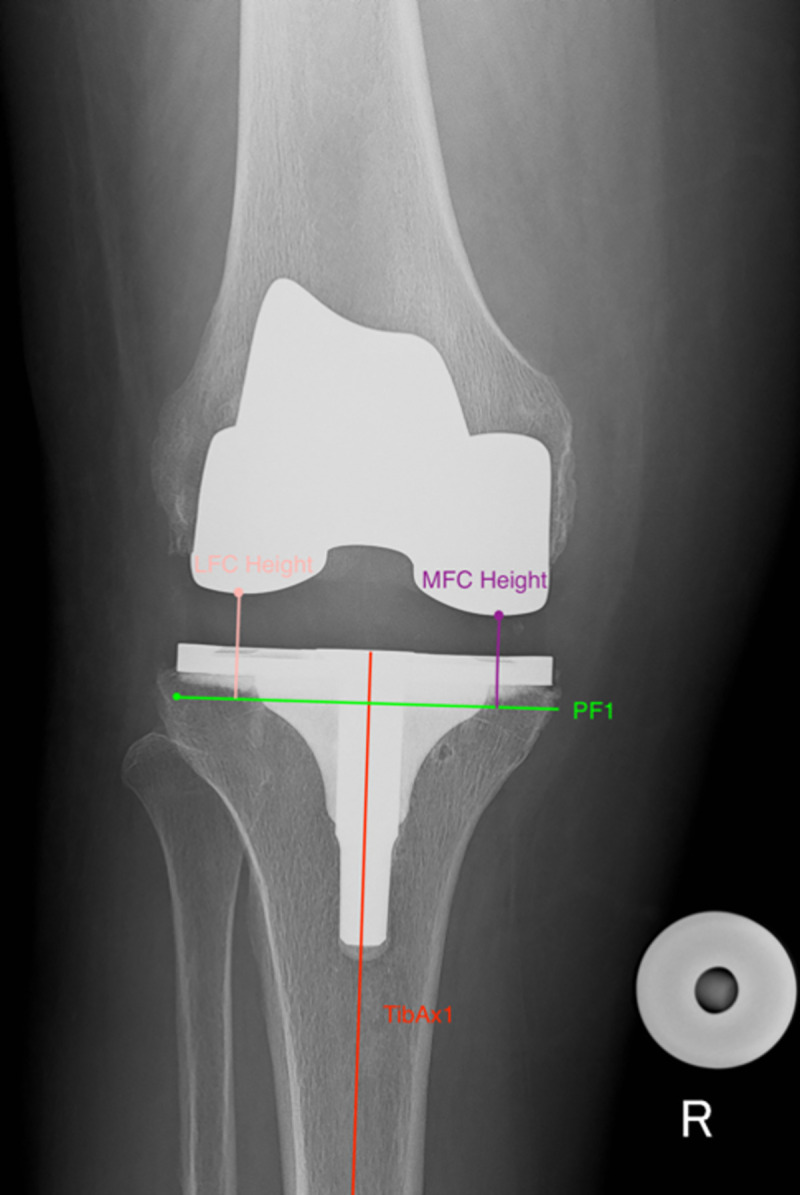
Post-operative IJLCM technique. Assessment of post-operative joint line height calculated as the average value of the Lateral Femoral Condyle (LFC) Height and the Medial Femoral Condyle (MFC) Height.

Post-operative Joint Line Height was also measured using the IJLCM (Fig 2): Measurements were performed on calibrated post-operative weightbearing AP. TibAx1 and PF1 were drawn using the same technique described for pre-operative radiographs. *Medial Joint Line Height*: the length of a line (parallel to TibAx1) from the most distal point on the medial femoral condyle to PF1 was measured. *Lateral Joint Line Height*: the length of a line (parallel to TibAx1) from the most distal point on the lateral femoral condyle to PF1 was measured. Varus/valgus cuts and asymmetrical polyethylene components were accounted for by calculating an average value of the medial and lateral joint line height.

The difference between the pre- and post-operative joint line height was established.

### Formulae for the IJLCM technique

$$\begin{array}{l} Pre‐operative\;joint\;line\;height\;(mm)=\\ \frac{Tibial\;Height\;(on\;less\;affected\;side)+Femoral\;Height\;(on\;less\;affected\;side)}{2}\\ Post‐operative\;joint\;line\;height\;(mm)=\\ \frac{Medial\;joint\;line\;height+Lateral\;joint\;line\;height}{2}\\ Change\;in\;joint\;line\;height\;(mm)=\\ Post‐operative\;joint\;line\;height–pre‐operative\;joint\;line\;height \end{array}$$

### Statistical analysis

All data were analysed using Statistical Package for the Social Sciences (SPSS, Version 26 IBM Corp, 2019. IBM SPSS Statistics for Windows, Armonk, NY: IBM Corp). All data sets were initially found to be normally distributed through a Shapiro-Wilk test. Average values, standard deviations, variance and margin of error of radiological measurements were calculated by each observer using each technique over two attempts. Inter-rater and intra-rater reliability were assessed for each measurement technique using an intra-class correlation coefficient (ICC). Cronbach alpha values for reliability were also tabulated. Box plots were plotted to appreciate the comparison between both measuring techniques. 2-tailed paired student’s T-test, analysis of variance (ANOVA) and ICC assessed the differences between measurements performed by senior orthopaedic surgeons and medical students.

## Results

**Fig 3:**
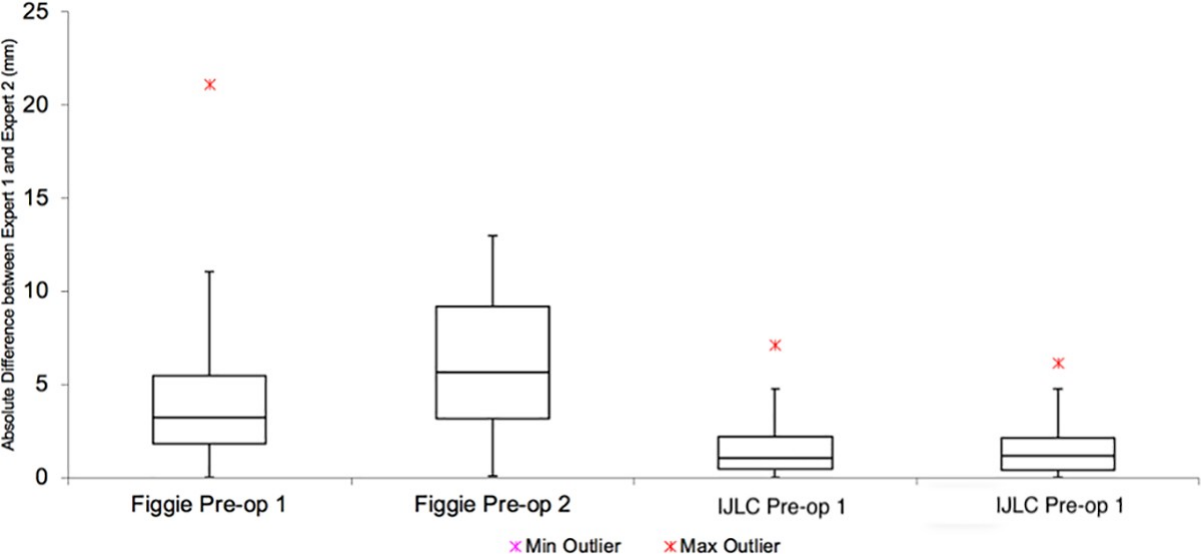
Boxplot demonstrating the differences between measurements performed by senior orthopaedic surgeon 1 and 2 on pre-operative radiographs.

**Fig 4:**
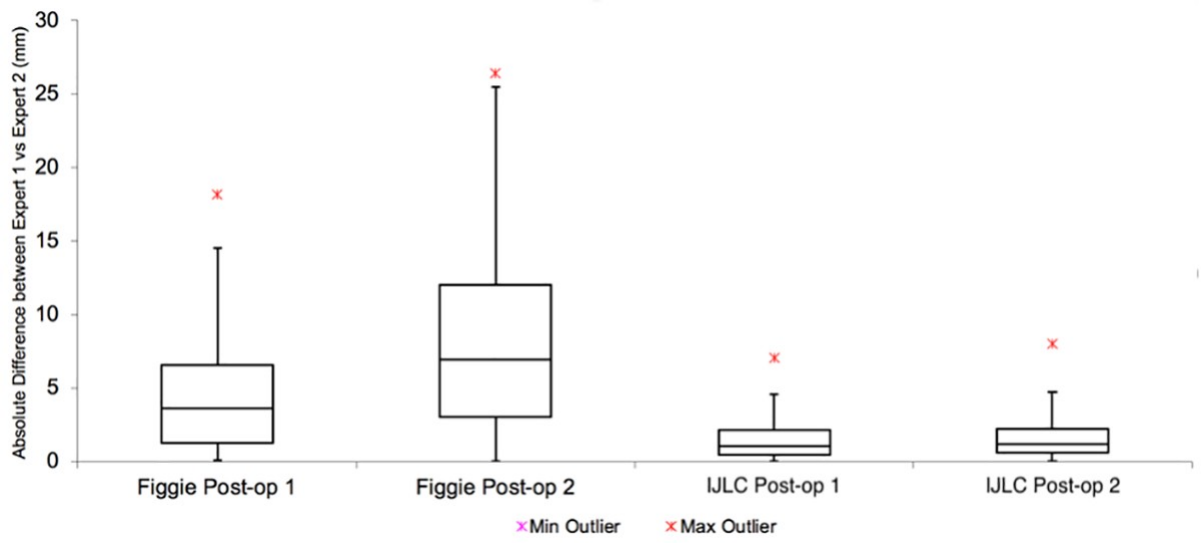
Boxplot demonstrating the differences between measurements performed by senior orthopaedic surgeon 1 and 2 on post-operative radiographs.

A total of 120 pre-operative and post-operative radiographs were included in this study. As shown in Tables 1 and 2, and demonstrated graphically in Figs 3 and 4, the difference in measurements between senior orthopaedic surgeons was smaller using the IJLCM. Table 3 demonstrates that IJLCM has a higher inter and intra-rater reliability between observers. When assessing intra-rater difference in measurements, 67 (55.8%) patient’s radiographic measurements were within 1mm on pre-operative images using the IJLCM. 95 (79.2%) were within 2mm and 112 (93.3%) were within 4mm using the IJLCM. In comparison, 9 (7.5%) were within 1mm, 23 (19.2%) were within 2mm and 36 (30.0%) were within 4mm when using the control technique. Similar differences are seen when looking at agreement on post-operative radiographs (56 (46.7%) <1mm, 82 (68.3%) <2mm and 109 (90.8%) using IJLCM versus 14 (11.7%) <1mm, 20 (16.7%) <2mm and 39 (32.5) using the control technique. Similar differences exist when comparing inter-rater differences in measurement.

**Table 1:** Analysis of differences in pre-operative joint line height measurements performed by senior orthopaedic surgeons.

	IJLCM		Control Technique	
	Difference (mm)	Absolute Difference (mm)	Difference (mm)	Absolute Difference (mm)
**Mean (SD)**	0.76 ± 2.70	1.83 ± 2.13	-1.46 ± 6.17	4.95 ± 3.93
**P-value**	0.20		0.04	

**Table 2:** Analysis of differences in post-operative joint line height measurements performed by senior orthopaedic surgeons.

	IJLCM		Control Technique	
	Difference (mm)	Absolute Difference (mm)	Difference (mm)	Absolute Difference (mm)
**Mean (SD)**	0.27 ± 2.51	1.65 ± 1.91	-0.53 ± 7.33	5.75 ± 4.54
**P-value**	0.85		0.53	

**Table 3:** ICC of measurements performed by senior orthopaedic surgeons.

	IJLCM		Control Technique	
	Pre-op	Post-op	Pre-op	Post-op
**Inter-rater ICC**	0.95 (0.93–0.97)	0.96 (0.95–0.97)	0.633 (0.508–0.726)	0.778 (0.682–0.845)
**Crohnbach Alpha**	0.95	0.96	0.634	0.778
**Intra-rater ICC**	0.92 (0.88–0.94)	0.97 (0.95–0.98)	0.501 (0.288–0.651)	0.581 (0.401–0.708)
**Crohnbach Alpha**	0.92	0.97	0.512	0.592

Table 4 demonstrates that medical students were able to achieve a high level of inter and intra-rater reliability with the IJLCM.

**Table 4:** Comparison of measurements performed by Medical Students (MS) and Orthopaedic Surgeons (OS).

Measurement	ANOVA (all 4 observers)	ICC (all 4 observers)	MS vs OS	MS1 vs MS2
**Pre-op 1**	0.42	0.92 (0.87–0.95)	0.13	0.69
**Pre-op 2**	0.83	0.92 (0.87–0.95)	0.47	0.58
**Post-op 1**	0.14	0.96 (0.92–0.98)	0.12	0.97
**Post-op 2**	0.40	0.96 (0.93–0.98)	0.33	0.87

## Discussion

Mean absolute difference between measurements performed by two senior orthopaedic surgeons using the IJLCM was 1.83mm on pre-operative images and 1.65mm on the post-operative images. The new technique has a high inter and intra-rater reliability (≥0.92) for pre-operative and post-operative image analysis. This suggests a high level of precision for the new technique.

The difference between measurements using the control technique [10] was significantly higher on pre-operative and post-operative measurements (p<0.01). Variance was smaller for the IJLCM, indicating a higher level of precision and accuracy.

Kawamura et al. [11] were the first to suggest measuring the distance between the tip of the fibula styloid and the joint line to identify the joint line height. The technique was later expanded upon by Snider et al. [12]. The measurement technique involved drawing a line from the superior aspect of the fibular head to the inferior aspect of the femoral condyles on an AP radiograph. Snider et al. [12] explained that the pre-operative joint line height should be an average of the joint line measurements of the medial and lateral compartments. The authors justified this claim by demonstrating that average values correlated most closely (ICC of 0.470) with the measurements performed using the control technique [10]. No previous validation of the control technique had been performed.

Snider et al. [12] claim that there was no statistically significant difference in functional outcomes in patients with variable joint line elevation. A significant limitation to this study was the lack of calibration between pre-operative and post-operative radiographs to correct for any magnification differences. Correcting magnification would likely have a significant impact on the accuracy of joint line measurements performed.

Further studies have been performed to assess the change in joint line height for primary and revision TKA. Jawhar et al [14]. presented a single-institution case-controlled study involving 493 patients. Measurements were performed using the technique described by Snider et al. [12]. Inter and intra-observer reliability was assessed on measurements of 30 cases. The study used a measurement tool with an accuracy of only 1mm. The authors reported an average joint line height change of 0.6mm, an intra-observer reliability of 0.87 and inter-observer reliability of 0.94. The width of the tibia was used to correct for magnification error between pre- and post-operative radiographs, however, this value can vary with rotation.

Babazadeh et al. [13] measured change in joint line height using long leg AP radiographs. The authors used the technique described by Snider et al. [12]. Magnification error was corrected on post-operative radiographs using the length of the tibial keel. Magnification between pre-operative and post-operative radiographs. A single observer performed 3 separate measurements of joint line height on their sample of 115 patients achieving an intra-rater reliability of 0.8.

This study measured the pre-operative joint line height from the least worn compartment as this best represents the patient’s pre-pathology anatomy. The pre-operative joint line height was determined as an average of the distance between the distal femur and tibial plateau (4.39mm) to account for cartilage thickness. The authors of this study accept that an assumption is made that the thickness of the cartilage is equal on the femoral and tibial side of the compartment. We suggest that the joint line lies in a region between the femur and tibia and the thickness of the cartilage should not be dismissed.

The seminal work by Figgie et al. [10] demonstrated an average change in joint line of 8.9mm following primary conventional TKA. In cases where joint line height was maintained within 8mm, no patients required manipulation, revision or suffered from patellofemoral pain. This is not consistent with findings demonstrated in subsequent series [13–15, 19]. The medial tibial plateau, lateral tibial plateau and tibial spines overlap on lateral radiographs and identifying the joint line accurately on a lateral radiograph can be challenging. Identifying a single point to reliably represent the tibial tuberosity is intuitively difficult. The difference in measurements was larger on post-operative images when using the control technique. A high variability in identification of the tibial tuberosity is suggested as the reason for this unexpected finding.

Comparison of measurements performed by medical students and orthopaedic surgeons using ANOVA, t-tests and ICC demonstrated good agreement. This suggests that the IJLCM can be used reliably by medical students to measure joint line height. Differences in measurement values between medical students were not statistically significant.

The limitations of our study include the use of short-leg AP and lateral radiographs; however, short-leg radiographs are more readily available in clinical practice. Additionally, with radiographs used from 6 different institutions, the protocol for performing plain radiographs varied. All images were reviewed by both observers and any cases where enough of the tibial metaphysis and diaphysis were not visible to identify the longitudinal axis were excluded. All pre-operative images required a calibration disc. Correction for magnification differences between pre- and post-operative radiographs was performed using the keel of tibial component. The length of the keel on a 2-dimensional image can change if there is a rotational error. Previous studies state that the use of the keel is more reliable than using bony landmarks [13].

## Conclusion

The relatively poor reliability of measurement techniques used when investigating change in joint line height make it difficult to compare results between studies. The IJLCM has been shown to have excellent inter and intra-rater reliability among orthopaedic surgeons and medical students, as well as better accuracy and precision than the techniques that have been used previously.

## Supporting information

S1 Data (XLSX)

## References

[1] Partington PF, Sawhney J, Rorabeck CH, Barrack RL, Moore J. Joint line restoration after revision total knee arthroplasty. In: Clinical Orthopaedics and Related Research. 1999. 10546611

[2] Martin JW, Whiteside LA. The influence of joint line position on knee stability after condylar knee arthroplasty. Clin Orthop Relat Res. 1990; 2208849

[3] Hofmann AA, Kurtin SM, Lyons S, Tanner AM, Bolognesi MP. Clinical and Radiographic Analysis of Accurate Restoration of the Joint Line in Revision Total Knee Arthroplasty. J Arthroplasty. 2006;21(8). doi: 10.1016/j.arth.2005.10.026 17162175

[4] Wyss TF, Schuster AJ, Münger P, Pfluger D, Wehrli U. Does total knee joint replacement with the soft tissue balancing surgical technique maintain the natural joint line? Arch Orthop Trauma Surg. 2006; doi: 10.1007/s00402-006-0171-0 16799793

[5] Ryu J, Saito S, Yamamoto K, Sano S. Factors influencing the postoperative range of motion in total knee arthroplasty. Vol. 53, Bulletin: Hospital for Joint Diseases. 1993. 8012266

[6] Singerman R, Heiple KG, Davy DT, Goldberg VM. Effect of tibial component position on patellar strain following total knee arthroplasty. J Arthroplasty. 1995; doi: 10.1016/s0883-5403(05)80210-4 9273377

[7] König C, Sharenkov A, Matziolis G, Taylor WR, Perka C, Duda GN, et al. Joint line elevation in revision TKA leads to increased patellofemoral contact forces. J Orthop Res. 2010;28(1). doi: 10.1002/jor.20952 19637213

[8] Insall J, Goldberg V, Salvati E. Recurrent dislocation and the high-riding patella. Clin Orthop Relat Res. 1972; doi: 10.1097/00003086-197210000-00012 5086583

[9] Yoshii I, Whiteside LA, White SE, Milliano MT. Influence of prosthetic joint line position on knee kinematics and patellar position. J Arthroplasty. 1991; doi: 10.1016/s0883-5403(11)80013-6 1875209

[10] Figgie HE, Goldberg VM, Heiple KG, Moller HS, Gordon NH. The influence of tibial-patellofemoral location on function of the knee in patients with the posterior stabilized condylar knee prosthesis. J Bone Jt Surg—Ser A. 1986; 3745240

[11] Kawamura H, Bourne RB. Factors affecting range of flexion after total knee arthroplasty. J Orthop Sci. 2001; doi: 10.1007/s007760100043 11484119

[12] Snider MG, MacDonald SJ. The Influence of the Posterior Cruciate Ligament and Component Design on Joint Line Position After Primary Total Knee Arthroplasty. J Arthroplasty. 2009; doi: 10.1016/j.arth.2008.08.009 19027265

[13] Babazadeh S, Dowsey MM, Swan JD, Stoney JD, Choong PFM. Joint line position correlates with function after primary total knee replacement: A randomised controlled trial comparing conventional and computer-assisted surgery. J Bone Jt Surg—Ser B. 2011; doi: 10.1302/0301-620X.93B9.26950 21911534

[14] Jawhar A, Shah V, Sohoni S, Scharf HP. Joint line changes after primary total knee arthroplasty: Navigated versus non-navigated. Knee Surgery, Sport Traumatol Arthrosc. 2013; doi: 10.1007/s00167-013-2580-2 23794005

[15] Goh GSH, Bin Abd Razak HR, Tan JYW, Yeo SJ. Intraoperative Measurements of Joint Line Changes Using Computer Navigation Do Not Correlate With Postoperative Radiographic Measurements in Total Knee Arthroplasty. J Arthroplasty. 2017;32(1). 10.1016/j.arth.2016.06.018 27430184

[16] Bin Abd Razak HR, Pang HN, Yeo SJ, Tan MH, Lo NN, Chong HC. Joint line changes in cruciate-retaining versus posterior-stabilized computer-navigated total knee arthroplasty. Arch Orthop Trauma Surg. 2013;133(6). doi: 10.1007/s00402-013-1738-1 23589064

[17] Pang HN, Yeo SJ, Chong HC, Chin PL, Chia SL, Lo NN. Joint line changes and outcomes in constrained versus unconstrained total knee arthroplasty for the type II valgus knee. Knee Surgery, Sport Traumatol Arthrosc. 2013;21(10). doi: 10.1007/s00167-013-2390-6 23322268

[18] Jawhar A, Hutter K, Scharf HP. Are joint line changes after primary navigated total knee arthroplasty predictable? J Orthop Sci. 2015;20(1). doi: 10.1007/s00776-014-0647-7 25217136

[19] Goh GSH, Liow MHL, Lim WSR, Tay DKJ, Yeo SJ, Tan MH. Accelerometer-Based Navigation Is as Accurate as Optical Computer Navigation in Restoring the Joint Line and Mechanical Axis After Total Knee Arthroplasty. A Prospective Matched Study. J Arthroplasty. 2016;31(1). doi: 10.1016/j.arth.2015.06.048 26220102

[20] Malghem J, Maldague B. Depth insufficiency of the proximal trochlear groove on lateral radiographs of the knee: Relation to patellar dislocation. Radiology. 1989;170(2). doi: 10.1148/radiology.170.2.2911676 2911676

